# Supplementary material for: Are carbon emissions trading and green financial instruments synergistic? -Comprehensive quantitative research based on content analysis
Source: PLoS One. 2024 Mar 7;19(3):e0298601. doi: 10.1371/journal.pone.0298601 (PMC10919601; doi:10.1371/journal.pone.0298601)
Supplement: S1 Data — (ZIP) [file pone.0298601.s001.zip › Data/地级市Z_F工作报告.zip!/║╙─╧/╜╣╫≈╩╨/└┤╫╘╣1⁄2╓┌║┼í╛┬φ┐╦╩2╛▌═°í┐_2019.docx]

各位代表：

现在，我代表市人民政府，向大会报告工作，请予审议，并请各位政协委员和列席人士提出意见。

一、2018年工作回顾

过去的一年，面对严峻复杂的国际国内环境和艰巨繁重的改革发展任务，全市上下以习近平新时代中国特色社会主义思想为指导，在省委、省政府和市委的坚强领导下，全面落实党的十九大精神和习近平总书记视察指导河南时的重要讲话精神，坚持稳中求进工作总基调，以新发展理念为引领，以高质量发展为根本方向，以供给侧结构性改革为主线，持续打好“四张牌”，围绕建设全面体现新发展理念示范城市，持续深入开展“质量、效率、法治、出彩”落实年活动，迎难而上、扎实工作，全市经济社会发展保持了“稳、进、好”的态势。综合实力稳步增强。地区生产总值增长6.3%，总量达2371.5亿元。结构调整深入推进。服务业增加值占地区生产总值比重达37.7%，同比提高1.9个百分点。高新技术产业增加值增长11%，高于规模以上工业增加值增速4个百分点。常住人口城镇化率达59.4%，居全省第四位，提高1.4个百分点。质量效益持续提升。一般公共预算收入增长8.8%，税收收入增长18.7%，提高6.4个百分点。税收占比达70.3%，创近10年来最好水平。规模以上工业企业利润增长12.9%。PM10、PM2.5浓度分别下降7.2%、8.2%，下降率均居全省第三位；省控目标断面水质达标率达95.8%，居全省第一位。人民生活不断改善。民生支出208亿元，增长12.9%，占一般公共预算支出77.3%。城乡居民人均可支配收入分别增长7.8%、8.7%。省、市重点民生实事圆满完成，社会大局和谐稳定。

一年来，经济社会发展取得了一系列新成就：一是着力提升品质内涵，擦亮了城市品牌。深入开展“四城联创”，实施主次干道维修、老旧小区改造提升、背街小巷整治、农贸市场提升等工程，扎实开展志愿者服务，城市承载能力、治理水平和文明程度全面提升，成功创建全国文明城市提名城市、全国水生态文明城市。二是聚焦发力项目建设，筑牢了发展支撑。坚持项目为王，强力推进“双十工程”，蒙牛乳业液态奶、河南超威正效一期等项目投产运行；南水北调城区段绿化带绿化部分基本完成，焦作火车站南广场、南站房建成投用，城市建成区黑臭水体基本消除，苏蔺水厂一期通水，中原路全线贯通，做实了产业转型升级、结构优化调整、加快高质量发展的抓手。三是抢抓新发展战略，提升了区域地位。全力打造中原城市群和豫晋交界地区的区域性中心城市、郑州大都市区门户枢纽城市，认真落实省委、省政府郑焦深度融合发展战略，围绕在中原更加出彩中出重彩、更精彩的目标，明确了建设“一枢纽、一中心、两新城、两走廊、三平台、四绿带”发展格局，构建高铁枢纽取得重大突破，南部产业新城蓬勃发展，北部生态新城有序铺开，郑焦综合交通体系全面推进，平台建设在创新中不断加强，郑焦深度融合全面提速，战略框架支撑显著增强，在中原城市群中的区域地位加快提升。四是狠抓重点突出特色，创造了“焦作经验”。“两定制兜底线”健康扶贫新模式受到国务院通报表扬。“两延伸、三提升、一集中、三聚焦”“四医联动”综合医改经验在全省推广。成为全省“一网通办”前提下的“最多跑一次”改革试点市。成功创建全国首家市、县、乡、村“四级联创”的国家级行政审批服务标准化试点市。全省综合治税、电商扶贫、旅游信息等现场会在焦召开，农村电商扶贫经验在全国电商论坛上推广，“焦作经验”叫响全国全省。五是准确把握重大机遇，实现了借力发展。超前谋划、精心运作，成功创建国家北方地区冬季清洁取暖试点城市、全国第三批山水林田湖草生态保护修复试点、全国重点采煤沉陷区综合治理试点、全国基层中医药工作先进单位等一批高含金量的“国家级牌子”，争取了一大批政策和资金支持，增强了我市可持续发展的后劲。通过一年来的努力，市十三届人大一次会议确定的目标任务有效完成，全市正在步入高质量发展的康庄大道。

主要做了以下六个方面的工作：

（一）聚焦发力关键环节，经济保持平稳增长。围绕关键重点，精准精确施策，有效破解难题，全市经济平稳运行、稳中向好。一是扩大有效需求。发挥投资关键作用，加强项目谋划储备，健全领导分包、观摩评价、集中开工等机制，转型发展攻坚项目库入库项目和累计新开工项目数量分别居全省第1位、第2位；省市重点项目完成投资分别占年度计划的128.3%、160.7%。着力培育消费热点，社会消费品零售总额增长10.9%，居全省第3位。持续加大力度，引进省外境内项目数量、进出口总额均居全省第3位。二是壮大实体经济。推动政策措施向实体经济倾斜，出台支持制造业高质量发展十条意见、促进民营经济高质量发展“3+N”等系列政策措施，实施放宽市场准入、降低企业成本等专项行动，减免企业增值税14.7亿元、小微企业所得税6170万元，帮助企业轻装上阵，有效激发了实体经济活力，全市新增市场主体4.2万户，同比增长8.1%。三是优化营商环境。深入开展“转变作风抓落实、优化环境促发展”活动，完善“六本台账”，加强“四项对接”，为重点企业解决融资150亿元、用工2.1万人、建设用地7706亩，有力保障了企业需求。启动优化营商环境三年行动计划，市直行政权力事项取消下放178项、调整356项，审批服务事项网上可办率、一网通办率、办件总量和人均办件量均居全省前列，为企业发展营造了宽松高效的环境。

（二）加快产业转型升级，供给质量持续提升。深化供给侧结构性改革，加快调整产业、产能、产品结构，供给质量效率不断提升。一是强力推进工业转型。加快建设中原经济区高端制造业先进基地，制造业十大产业增加值增长9.1%，高于规模以上工业增加值增速2.1个百分点。全面开展“三大改造”，417个项目完成投资345亿元，中原内配等企业获评“绿色工厂”。深入实施大企业集团培育工程，蒙牛产业集群主营业务收入突破百亿元，超百亿、超50亿企业分别达2家、12家。强力推进210个重大工业转型升级项目，完成投资299亿元。启动百企退城三年行动计划，搬迁改造企业68家、完成20家。实施“千企上云”行动，培育两化融合管理体系贯标企业19家、对标企业156家。化解煤炭过剩产能120万吨。二是大力发展现代服务业。加快建设国际文化旅游名城，云台古镇、嘉应观田园综合体等项目顺利实施，云台山被评为省三钻级智慧景区，青天河、神农山被评为省一钻级智慧景区，云台山旅游节荣获纪念改革开放40周年“中国优秀节事奖”，我市被评为中国体育旅游十佳精品目的地，旅游综合收入增长12.3%。围绕打造中原经济区文化康养业引领基地，加快建设陈家沟太极产业新城，强力推进云阶康养小镇、保和堂（焦作）产业园等重大康养业项目，与张艺谋团队成功签约，启动《印象·太极》大型实景演艺项目；与阿里巴巴实现战略合作，共同打造集竞技比赛、体育健身和衍生产业于一体的功守道赛事。电商交易额达911亿元，增长23.2%。驮丰多式联运物流港等项目加快建设，鹏海保税博览城保税仓库一期建成投用，新增A级物流企业2家、省级现代服务业专业园区1个，获评全国供应链创新与应用试点城市。以“政府主导、信用为基、服务为本、智于解困、慧于创新”为原则，建成全国领先、全省首家智慧金服平台，注册企业1103家，实现放款108亿元，打造了破解中小微企业融资难、融资贵问题的“焦作模式”。新增新三板挂牌企业1家、四板挂牌企业64家，新引进金融机构5家。房地产市场保持平稳健康发展。三是加快发展载体建设。孟州市、武陟县产业集聚区考核综合排序分别居全省第8、第11位，孟州市产业集聚区晋升三星；解放区特色商业区晋升三星，修武县、中站区特色商业区晋升一星，发展载体建设持续走在全省前列。

（三）强力开展三大攻坚，瓶颈制约加快破解。坚持把打好打赢三大攻坚战作为破解瓶颈制约、全面建成小康社会的硬任务，系统谋划、强力推动，取得了阶段性重大成果。一是防范化解重大风险。认真防范化解政府债务风险，严格控制增量，稳妥化解存量，全市政府债务率稳步下降。积极化解企业风险，重点企业资产负债率持续下降。出台银行业自律公约，妥善处置金融风险点，维护了金融秩序和金融稳定。二是扎实开展脱贫攻坚。制订打好精准脱贫攻坚战三年行动计划，组织开展“四场硬仗”“六大行动”和“四项工程”，攻坚任务扎实推进。实施产业扶贫项目167个，建成就业基地62个，培训贫困家庭劳动力1.2万人，56个贫困村、1.31万人实现脱贫。结对帮扶嵩县工作全面推进。三是大力推进污染防治。出台坚决打好污染防治攻坚战的实施意见，强化“六控”措施，调整“四个结构”，350家企业实现提标改造，完成11台65蒸吨及以下燃煤锅炉拆改，完成“双替代”改造9.2万户，环境空气质量持续改善。各级河长累计巡河6万余次，实施城区河道综合整治、畜禽养殖污染治理等工程，清理河道816公里。出台土壤污染防治攻坚“1+3+9”制度体系，土壤污染状况详查扎实推进，土壤环境质量总体保持平稳。启动国土绿化提速、建设森林焦作行动，完成森林资源培育19.2万亩，建成公路绿色廊道306公里，森林覆盖率持续提升。

（四）深化改革开放创新，发展动能有效激活。坚持以改革增动力、以开放借外力、以创新添活力，为高质量发展积蓄了强劲动能。一是坚定不移推进改革。深化国有企业改革，基本完成省定30家“僵尸企业”处置任务，化解债务6.9亿元，直接安置职工2450人，全面打赢“僵尸企业”处置总攻战。深化医药卫生体制改革，在全省率先推进公立医院管理体制改革，基本实现区域内医联体全覆盖、医联体远程诊疗全覆盖。农村土地承包经营权确权登记颁证、国有林场改革基本完成。新的市与县（市）区财政体制运行成效显著，机构改革和国土、林业、电力、公共资源交易等改革扎实开展。二是持续扩大对外开放。组团参加进博会、广交会、东博会等重大招商节会，赴长三角、珠三角、京津冀、青岛等地举办系列推介招商活动，引进省外境内项目438个，合同利用省外资金1377亿元，实际到位资金658亿元。焦作进口肉类指定查验场投运，全市进出口总额达161.5亿元，其中出口达117.3亿元。三是强化科技创新驱动。主动融入郑洛新国家自创区，6家单位被确定为自创区首批辐射点。河南理工大学科技园获批省级大学科技园，入驻企业40家。新增高新技术企业30家、总数突破100家，新增省工程技术研究中心13家、省院士工作站1家，多氟多获批设立国家博士后科研工作站。健全“引才聚焦”人才政策体系，引进高层次紧缺人才2123人，评选全市第二批创新创业领军人才（团队）13个，落实扶持资金1850万元。研发与试验经费投入强度居全省第6位，获得授权专利增长34.4%，万人有效发明专利拥有量5.16件，居全省第3位。发放科技贷款1.05亿元，成为全省首批科技与金融结合试点市。

（五）扎实推进城乡建设，协调发展步伐加快。统筹实施乡村振兴战略和城市转型发展，“城乡一体、四级联动”的新型城镇化发展格局加快构建。一是全面改造提升中心城区。以“四城联创”为载体，以十大基础设施项目为龙头，深入开展中心城区改造提升行动，实施道路维修项目40个，整治社区楼院612个、背街小巷156条，新增供热面积270万平方米，垃圾、污水处理率分别达100%、95%。二是强力推进百城建设提质。统筹推进百城建设提质和文明城市创建，实施提质项目449个，设立500万元奖补资金，建立观摩点评、专项督导、考核激励等机制，百城建设提质工作在全省作经验介绍。三是深入实施乡村振兴战略。夏粮单产全省第一，在全省粮食安全行政首长责任制考核中获得优秀等级。加快推进“四优四化”，建成12个省级农业产业化联合体，农业产业化集群销售收入716亿元。农民合作社、家庭农场分别达5838家、1028家。温县获批国家现代农业产业园。在全省率先编制完成乡村振兴战略规划，扎实开展农村人居环境整治，农村住房、供电、厕所等建设全面提速，“互联网+农业”、生态农业、乡村旅游、农村电商等新业态蓬勃发展。建成“四好农村路”1054公里。孟州源沟村被评为中国美丽休闲乡村。

（六）着力增进民生福祉，社会大局和谐稳定。坚持以人民为中心的发展思想，公共服务水平和均等化程度持续提升，群众获得感、幸福感、安全感不断增强。一是稳定扩大社会就业。开展重点企业专场招聘会、就业岗位推荐专项行动，建成全省首家市级农民工综合服务中心，新发放创业担保贷款5亿元，城镇新增就业7.55万人，新增农村劳动力转移就业3.07万人。二是加快发展公共事业。新建改扩建幼儿园37所、中小学73所，实现国家义务教育发展基本均衡县（市）区全覆盖。深化职业教育“双元制”改革，产教融合、校企合作深入推进。高考一本上线5190人，同比增长20%。加快乡镇卫生院、村卫生室标准化建设，15分钟卫生服务圈基本形成。扎实开展文化惠民工程，累计建成基层综合性文化服务中心1751个，百姓文化超市入选国家公共文化服务体系示范项目创建名单。中国老年人体育协会太极拳专项委员会落户焦作。十三届省运会综合成绩位居前列，省第七届残运会上取得金牌、奖牌、总分三项第一。成功创建全省首家省级示范数字档案馆。三是完善社会保障体系。提高退休人员养老保险待遇，出台助保贷款实施办法，城乡居民养老参保率达到99.5%。全面推动医保支付方式改革，异地就医直接结算范围持续扩大。在全省率先开展贫困精神残疾人集中托养工作。棚改安置房开工2.9万套，公租房分配3.2万套，住房保障水平持续提升。四是加强社会治理创新。完善社区治理体系，基本完成“334”楼院协商治理模式推广工作。沁阳邘邰村、博爱大新庄被评为全国民族团结进步创建示范区。深化平安焦作建设，严格落实安全生产责任，全面加强食品药品安全监管，依法做好信访稳定工作，完善社会治安防控体系，深入开展扫黑除恶专项斗争，公众安全感指数居全省前列。妇女儿童、残疾人、红十字、慈善等事业健康发展，审计、外事侨务、统计调查、对台、史志、气象、地震、人防、援疆等工作都取得了新的成绩。

一年来，我们自觉接受人大法律监督、工作监督和政协民主监督，依法依规向人大及其常委会报告工作、向政协通报情况，认真办理人大代表议案、建议和政协提案；我们主动接受社会监督和舆论监督，有效改进和推进了政府工作。

各位代表，这些成绩的取得，是以习近平同志为核心的党中央英明领导的结果，是省委、省政府和市委正确领导的结果，是市人大、市政协、焦作军分区大力支持的结果，是全市人民拼搏奋斗的结果。在此，我代表市人民政府，向辛勤工作在各条战线的干部群众，向人大代表、政协委员，向各民主党派、工商联和无党派人士、各人民团体，向驻焦部队、武警官兵和公安干警，向离退休老领导、老同志，向关心支持焦作发展的社会各界人士，表示衷心的感谢并致以崇高的敬意！

各位代表，回顾一年来的奋斗历程，我们深切地感受到：一是必须坚持党的集中统一领导。我们坚持以习近平新时代中国特色社会主义思想为指导，树牢“四个意识”、坚定“四个自信”、做到“两个维护”，坚持以政治建设为统领，面对经济下行压力持续加大、多重约束愈加趋紧、转型升级困难增多等复杂形势，将党的领导贯穿到政府工作的全过程、各环节，牢牢抓住发展第一要务，紧紧围绕党中央的决策部署寻求破解新矛盾、新问题的新方法、新举措，抓重点、补短板、强弱项、稳增长，牢牢把握了发展主动权。实践证明，有以习近平同志为核心的党中央掌舵领航，无论风浪多大，中国经济这艘大船都能沿着正确方向行稳致远。二是必须全面深化改革。我们积极响应习近平总书记“将改革进行到底”的号召，在承接上级改革任务的同时，进一步注重从群众关注的“焦点”、经济发展的“堵点”中寻找改革突破点，全面深入推进各项改革。新的市与县（市）区财政体制改革调动了各方积极性，健康扶贫、四医联动等综合医改惠及广大群众，智慧金服改革赢得企业、金融机构纷纷点赞，市县乡村“四级联动”的行政审批改革真正方便了企业和群众，从而激发了市场主体的内在动力。实践证明，发展中的矛盾和问题，唯有靠改革破解，改革就是解放和发展生产力。三是必须持续扩大开放。在全球化时代，在全面开放的市场经济条件下，任何地方仅靠自身“滚雪球”，都不可能实现跨越发展，无论发展存量，还是培育增量都必须全方位扩大开放，全力开展招商引资引智。去年，我们不断在扩大开放上下真功、在招商引资上出实招，顺利实现了“六稳”，保持了经济平稳增长。实践证明，必须借开放调整结构、做优存量，靠开放无中生有、做强增量，开放是决定前途命运的关键抉择、是发展的生命线，高水平开放是推动高质量发展的活水活力。四是必须厚植基础优势。我们强力推进“双十工程”，深入开展“四城联创”，扎实推动百城建设提质，加快构建现代产业体系，大力推进南北产业新城建设，谋划推进公铁及无水港综合交通运输体系，着力构建枢纽支撑，发力抢抓7座跨黄河大桥等综合交通项目，奋力争取呼南高铁豫西通道项目，这些工作不仅扩大了有效投资，为稳增长作出了贡献，更为焦作长远发展夯实了支撑。实践证明，多干利长远的实事，多做厚植优势的大事，秉承“功成不必在我”的精神，高质量发展才能奠定坚实基础。五是必须狠抓落实。习近平总书记强调，空谈误国，实干兴邦。我们持续深入开展“质量、效率、法治、出彩”落实年活动，扭住关键、精准发力，全面落实中央、省决策部署，推动经济发展步入高质量发展轨道。去年，三产占比、税收占比、企业利润等质效指标越来越好，政府系统行政效率、依法治理水平不断提升，许多工作走在全国全省前列。实践证明，一分部署，九分落实。落实才能出质量、出效率、出精彩。对于这些经验和体会，我们要按照省委营造“学的氛围、严的氛围、干的氛围”要求，在今后的工作中继续坚持和发扬。

在总结成绩的同时，我们也清醒地认识到，经济社会发展还有一些短板和不足。一是经济结构调整任务艰巨，传统产业转型步伐不快，先进制造业和现代服务业比重偏低，新产业、新业态规模仍然较小。二是科技创新能力不足，创新载体不多，创新机制仍然不活。三是中心城区首位度不高，承载力、吸引力、竞争力、带动力不强；城乡之间、县域之间发展仍不平衡。四是公共服务体系不完善，优质教育、文化、医疗、养老等服务供给与群众期盼仍有不小差距，环境污染防治任务依然艰巨。五是作风转变还不到位，有的干部思想观念、能力素质与高质量发展的要求还不适应。我们一定直面问题，采取有力措施，认真加以解决。

二、2019年工作安排

今年是新中国成立70周年，是全面建成小康社会、实现第一个百年奋斗目标的关键之年，做好今年工作意义重大。今年工作的总体要求是：坚持以习近平新时代中国特色社会主义思想为指导，以建设全面体现新发展理念示范城市为引领，深入贯彻中央经济工作会议、省委十届六次八次全会和市委十一届九次全会精神，以党的建设高质量推动经济发展高质量，全面实施创新驱动、开放带动、生态立市三大战略，加快建设创新、富裕、美丽、平安“四个焦作”，早日跻身全省“第一方阵”，致力打造精致城市、品质焦作，努力在中原更加出彩中出重彩、更精彩，以优异成绩庆祝新中国成立70周年。

今年经济社会发展主要预期目标是：生产总值增长7.5%—8%，一般公共预算收入增长7.5%，税收占一般公共预算收入的比重达到72%以上，固定资产投资增长8.5%左右，社会消费品零售总额增长10.5%以上，居民消费价格涨幅控制在3%左右，经济结构进一步优化，质量效益明显提升。全力推动创新发展，研究与试验发展经费投入强度达到1.7%，高技术产业、战略性新兴产业增加值占规模以上工业增加值的比重分别达到10%以上和20%左右。努力加快协调发展，常住人口城镇化率提高1个百分点以上，人均预期寿命持续提高，九年义务教育巩固率达到95%，医疗卫生、文化、体育等社会事业全面进步。坚决做到绿色发展，单位生产总值能耗下降3%以上，主要污染物排放量控制在省定目标以内。有效扩大开放发展，进出口保持平稳增长。持续提升共享发展，居民人均可支配收入增长8%，1.54万农村贫困人口脱贫，城镇新增就业6万人以上，城镇调查失业率和城镇登记失业率分别控制在5.5%以内、4%以内。这些预期目标，更加凸显了新发展理念和高质量发展要求，有利于把工作重点放在推进高质量发展上来。

实现这些目标，必须做到“五个坚持”：一是坚持以实体经济强基固本。工业是我市实体经济的核心，认真落实实施创新驱动发展战略进一步推进“双创”深入发展的意见、支持制造业高质量发展十条意见，围绕十大先进制造业，制订年度行动计划，强力推进产业发展，夯实稳定增长的基础。二是坚持以基础建设提升品质。以“四城联创”、中心城区改造提升、百城建设提质和乡村振兴战略实施为抓手，提升文明素质，完善基础设施，增强城市功能，促进区域协调，打造精致城市、品质焦作。三是坚持以新城创建积蓄动能。准确把握南部产业新城、北部生态新城的产业定位，主动承接郑州等地先进产业转移，做实郑焦深度融合主阵地，打造焦作出彩发展新的增长极。四是坚持以改革开放优化环境。持续推动财税、金融、市场准入、行政审批等改革走向深入，进一步敞开开放大门，不断完善政策支撑、载体平台和服务体系，全面优化营商环境。五是坚持以共建共享改善民生。对标全面小康目标，着力补齐教育、医疗、文化、社保等民生短板，全面提升基本公共服务供给能力，让全市人民群众的生活一年更比一年好。

重点做好以下八个方面的工作：

（一）着力稳定经济增长，夯实高质量发展基础。坚持精准出招应变，扶持实体经济，强化项目支撑，扩大市场需求，培育新的动能，保持和巩固经济稳中向好态势。

支持实体经济发展。强化要素资源保障，推动国家和省税费、用能、用地、融资、物流等领域降成本政策落地，进一步完善我市相关政策。实施优化营商环境三年行动，在企业开办和注销、施工许可证办理等方面，持续提升服务水平。抓好《焦作市促进民营经济高质量发展的实施意见》落地，完善全市“个转企”培育清单和激励制度，鼓励民营企业参与国有企业混合所有制改革，全面落实相关优惠政策，扶持民营企业加快发展。大力弘扬企业家精神、劳模精神和工匠精神，助推企业制度、科技、产品、模式创新，促进市场主体健康发展。

加大项目建设力度。完善项目观摩第三方评价机制，探索建立重大项目谋划专家咨询委员会，加强项目质效评估，促进投资结构优化调整。设立项目前期鼓励资金，紧盯国家、省政策导向，聚焦基础设施、三大改造、乡村振兴、生态环保、民生改善等重点领域，积极谋划争取一批重大项目。抓好总投资946亿元的132个市重点项目建设，确保年度投资超300亿元。强力推进“三区”建设，实施产业集聚区高质量发展专项行动，积极申报国家级战略性新兴产业集群，抓好配套设施完善、产业集群集聚等工作，确保主营业务收入增长8%以上；实施商务中心区提速、特色商业区提质工程，力争社会消费品零售总额增长11%以上。

激活消费市场需求。实施消费升级行动，完善提升万达广场、王府井、摩登街、大杨树等商业街区，积极创建省级步行街、省级品牌消费集聚区。依托鸿运国际商城、西部工业原料城，推进中心城区专业市场外迁。加快全市社区便民消费服务中心、农村生活综合服务中心、农产品流通站点等建设。深入推进商务综合监管执法体制改革，优化消费环境，激发消费潜力。

大力发展“三新经济”。聚焦大数据、云计算、工业物联网、工业设计、数字创意等新经济新业态新模式，再培育一批“三新”企业。建好工业互联网、工业云、工业大数据、分享经济、大企业“双创”等平台，推进焦作浪潮大数据产业园项目，打造河南工业互联网重点综合平台和大数据产业基地。推动300家企业“上云”，建设两化融合管理体系贯标企业20家、对标企业200家，确保两化融合发展指数稳居全省前列。

（二）贯彻落实“八字方针”，提升发展质量效益。按照“巩固、增强、提升、畅通”八字方针总要求，加快发展方式转变、经济结构转型、增长动能转换，不断提高经济发展质量和效益。

“巩固”去降补成果。制订铝工业、煤电、钢铁、水泥、煤化等重点行业三年转型行动方案和年度专项计划，压实工作责任，明确时间节点，细化转型任务，推动产能落后和过剩行业出清。抓好“僵尸企业”处置后续工作，做好债务化解、税收支持、资产处置等重点任务。严格“破立降”奖补资金拨付使用，落实好去产能职工安置、债务处置等政策，形成正向激励和优胜劣汰的导向。

“增强”微观主体活力。打好优化发展环境“组合拳”，营造便利高效政务环境、开放公平市场环境、公正透明法治环境、诚实守信信用环境。大力清理规范涉企收费项目，实行收费项目清单制，实现清单之外对企业“零收费”。深化“放管服”改革，深入推进“一网通办”前提下的“最多跑一次”改革，加强资源整合、流程优化、制度创新，提升政务服务大厅“一站式”功能，70%以上事项实现“一窗”分类受理，80%以上事项实现网上可办，100个高频事项实现“最多跑一次”，全力为市场主体打造优质高效的政务环境，让焦作政务服务品牌在全省更加出彩。

“提升”产业链水平。实施高端装备、新材料、新能源汽车、绿色食品、康养等十大优先发展产业专项行动计划，完善我市产业转型“时间表”和“路线图”，着力强链、补链、延链，构建完整的上下游全产业链，再打造一批集中度高、成长性好、竞争力强的产业集群。坚持围绕产业链布局创新链、安排资金链，围绕智能制造、汽车及零部件、现代化工、生物医药等支柱产业，组织实施一批重大科技攻关专项，解决好产业链延伸中存在的“卡脖子”问题。推动产业深度融合，在产业集聚区内规划建设一批生产性服务业配套园区，推动供应链、生产链、服务链相融合，打造全产业链经济。

“畅通”经济循环系统。开展重点领域综合金融服务行动，发挥智慧金服平台、科技支行等作用，通过线上线下互动互补，畅通金融服务实体经济的渠道。进一步完善土地、劳动力市场相关制度，引导要素资源向市场主体集聚，畅通市场配置要素的运行机制。实施市场准入负面清单制度，坚决破除要素流动壁垒，保障各类市场主体公平参与市场竞争。强化产销、产需、产运对接，加快推进“公转铁”，加快畅通流通循环系统，推动实现金融和实体经济、经济增长和就业扩大、市场和生产主体的良性循环。

（三）加快产业转型升级，强化高质量发展支撑。坚持以供给侧结构性改革为主线不动摇，深入开展产业转型攻坚，为高质量发展提供坚强产业支撑。

推动制造业高质量发展。坚持调整存量与优化增量并举，强力实施“四大工程”，着力打造先进制造业强市。实施传统优势产业改造提升工程。以智能制造引领“三大改造”，培育3个智能工厂、8个智能车间，支持中原内配等企业由智能车间向智能工厂升级；争取创建2家绿色工厂，1家企业进入国家能效水效“领跑者”名单；推动300家规上工业企业实施技术改造，技改投资占工业投资比重达到25%以上。统筹实施百企退城行动，完成45家搬迁任务。实施战略性新兴产业培育壮大工程。加快推进和光光电产业基地、同仁堂中药配方颗粒、亿水源铝酸钙粉等项目建设，大力发展电子信息、现代生物、新材料、新能源等战略性新兴产业，培育一批转型升级“头雁企业”。实施大企业集团培育工程。抓住省里支持大型制造企业发展重要机遇，扶持重点企业实施上下游产业链、左右岸配套项目，力争超50亿、超百亿企业实现新突破。同时，大力发展科技型中小微企业，加快打造一批专精特新“小巨人”和一批制造业单项冠军。实施重大项目引领工程。强力推进十大工业项目，全年完成投资50亿元。龙蟒佰利联钛锆产业园、隆丰皮草牛皮制品、蒙牛乳业核心工厂板块和配套包装及牧场、御捷新能源纯电动车产业园、河南超威动力型和储能型电池、中德高端汽车零部件产业园等投产或部分投产，多氟多锂离子动力电池一期、河南城盾智能盾构机及矿机设备产业园一期、蛮蛮云二期、国家智能清洁能源汽车质量监督检验中心一期等项目完成设备安装调试。同时，以十大工业项目为引领，加快建设57个重点工业项目，确保完成投资135亿元，为制造业高质量发展提供有力支撑。

促进服务业提质增效。坚持传统服务业和现代服务业并举，生产性服务业和生活性服务业并重，推动生产服务集聚化、生活服务便利化、基础服务网络化。促进旅游业转型升级。实施旅游十大创新引领工程，加快云台古镇、陈家沟旅游服务综合体等项目建设，推动云台山、青天河、神农山、陈家沟、嘉应观等景区提升发展，全力打造南太行生态旅游集聚带、黄河生态文化旅游带。完善中心城区旅游产品体系，加快泽盛煤场、百年电厂文旅园区等项目建设，实施南水北调生态休闲带、大沙河滨水景观带等重大文旅项目，支持蒙牛乳业创建AAAA级工业旅游景区。培育壮大文化康养业。加快推进陈家沟太极产业新城、云阶康养小镇、白鹭湖生态康养小镇、南太行国家级登山健身步道等项目，打造《印象·太极》大型实景演艺节目，办好功守道赛事，扎实推进河南太极拳学院项目建设，完善太极拳研学游、体验游、养生游等产品体系，推动太极拳世界申遗获得实质性突破，创建一批全国中医药健康旅游示范区和示范基地，加快建设“世界太极城·中原养生地”。创新发展现代物流业。围绕建设全国供应链创新与应用试点城市，引进一批包括供应链核心企业、服务企业和终端企业在内的供应链骨干企业，积极培育供应链、多式联运、物流金融等新业态，大力发展冷链物流、快递物流等特色物流。围绕构建郑焦晋物流走廊，编制郑焦物流融合发展规划，加快建设武陟公铁物流港、中国宝武焦作现代综合物流园、万邦国际农产品冷链物流园、日照港博爱无水港等项目，新增国家AA级以上物流企业8家。规范发展电商产业。建设电商大数据综合信息平台，支持百大超市等大型商贸企业开展网络批发和零售业务。加快建设电商物流村级配送点、快递服务点，推广“电子商务+生产基地+社区直配”生鲜直销模式。深化“丝路电商”国际合作，加快建设焦作跨境电商生态圈。加快发展现代金融业。深入实施引金入焦工程，大力发展基金、融资租赁、资产管理等现代金融业态。用好小微企业贷款风险补偿资金，加强社会信用体系建设，打造良好金融生态环境。完善智慧金服平台，加快发展普惠金融、科技金融、绿色金融，提高服务实体经济能力。完善企业上市奖励政策，加快推进云台山、科瑞森、永威安防、伊赛牛肉等企业上市，支持中旅银行围绕打造旅游特色赴港上市，加快培育一批科技型上市后备企业。积极推进农信社改制，全面完成县级农信社改制和焦作农商行组建工作。保持房地产业平稳健康发展。做好问题楼盘化解攻坚工作，完善住房市场和住房保障体系，扩大保障房覆盖面，确保房地产市场稳定。推动外地建筑业企业就地法人化，加快建筑业转型发展。

（四）打好打赢三大攻坚战，补齐高质量发展短板。把打好打赢三大攻坚战摆在全局更加突出的位置，全力攻坚、务求实效，确保完成年度攻坚任务。

全力防范化解风险。全面加强金融监管，发挥好企业债权人委员会作用，有效防控企业债券违约、担保圈、网络借贷、非法集资等风险隐患。探索开展民营企业债转股，引导企业加大股权融资力度，降低企业经营风险。出台防范化解政府隐性债务风险意见，科学安排年度预算资金，有效盘活存量资金，依法规范举债行为，有序稳妥化解政府隐性债务。树立底线思维，压实安全责任，全面推行安全生产风险隐患双重预防体系建设，整合消防、水旱和地质灾害防治等应急力量，健全应急管理体系；抓好交通、校园、建筑、食品药品、危险化学品等重点行业安全监管，坚决防范遏制各类重特大事故发生。

扎实推进精准脱贫。坚持精准方略，把提高脱贫质量放在首位，深入实施打赢脱贫攻坚战三年行动计划，继续打好“四场硬仗”、开展“六大行动”、实施“四项工程”，突出抓好产业扶贫、就业扶贫、金融扶贫、社会扶贫等重点工作，打好脱贫攻坚总攻战。建立脱贫正向激励和稳定脱贫长效机制，努力防止贫困人口返贫。全面推开贫困兜底线保障，启动兜底线贫困人口认定工作，对有意愿和需求的失智、失能、重残贫困对象实施集中托养，确保兜底线保障工作大头落地。加大结对帮扶力度，全面落实“四项协议”，助力嵩县如期脱贫。

强力开展污染防治。高质量完成中央环保督察“回头看”及省委、省政府大气污染问题专项督察整改工作，突出强治根、严执法，坚持点、线、面标本兼治，气、水、土同步联动，加快推动环境保护由“治”到“建”，确保完成全年目标任务。打好蓝天保卫战。持续抓好“六控”，优化调整“四个结构”，推进重点涉气企业特别排放限值改造，开展“散乱污”企业动态清零行动，加快国家北方地区冬季清洁取暖试点城市建设，完成既有建筑节能改造203万平方米，完成“双替代”15.13万户。加快人工影响天气作业示范基地建设。制定焦作市大气污染防治条例。煤炭消费总量控制完成省定任务。打好碧水保卫战。严格落实河长制，开展河道“清四乱”和河道提升工程，改善大沙河、共产主义渠、蟒河等河流环境流量。加快实施“四水同治”，积极推进南水北调城区段生态保护、九渡水库等“四水同治”十大工程。打好净土保卫战。加强重金属污染、农业面源污染防治和固体废物处理，抓好土壤污染管控和修复工作。深入推进全国山水林田湖草生态保护修复试点建设，扎实开展国土绿化提速、建设森林焦作行动，实施矿山地质环境治理、国储林基地等项目，完成森林资源培育16.75万亩，新增干线公路绿色廊道101公里。实施绿色建筑提升行动，建成东部商砼产业园，加快建设静脉产业园、装配式建筑产业园、西部商砼产业园。

（五）持续深化改革开放，激发高质量发展活力。加大力度推进改革，推动全方位高水平开放，以改革释放生产力，以开放增强竞争力，为高质量发展提供源源活力。

聚焦发力重点改革。深化国有企业改革。持续深化国企产权结构、组织结构、治理结构改革，试点推进混合所有制改革。组建国有资本运营（投资）公司，建立经营性国有资产统一监管体制。深化财税体制改革。加快构建现代预算制度，实施预算绩效管理，提高财政资金配置效率和使用效益。巩固提升新的市与县（市）区财政体制改革成果，设立财政体制改革调节基金，充分调动各方培植财源税源的积极性。深化投融资体制改革。用好用足地方政府债券政策，发挥好地方政府性融资担保体系作用，规范发展政府和社会资本合作项目。深化医药卫生体制改革。加快城市公立医院综合改革，健全现代医院管理制度，强化医疗卫生行业综合监管，规范医联体（医共体）建设，完善分级诊疗制度，深入推进医保支付方式改革，加强药品耗材供应保障，积极发展“互联网+医疗”健康服务新模式。坚持中西医并重，传承发展中医药事业，全面推进“健康焦作”建设。深化农村综合改革。继续推进农村承包地“三权分置”改革，完善县乡村三级农村土地流转服务网络，深入开展“空心村”治理，探索农村宅基地改革。深化农村集体产权制度改革，完成农村集体资产清产核资工作，推进集体资产股份合作制改革，发展壮大新型村级集体经济。同时，有序推进机构改革，统筹推进教育、文化和旅游、自然资源和规划、电力等领域改革。

强力推进对外开放。拓展开放领域，提高开放质量，建好开放平台，努力打造内陆开放新高地。高质量办好“一赛一节”。举办好中国焦作国际太极拳交流大赛暨云台山旅游节，让“太极圣地、山水焦作”品牌叫得更响。坚持节会搭台、经贸唱戏，高标准办好跨境投资与贸易对接会、专题招商推介会、怀药博览会等系列活动，提升节会综合效益。全力抓好招商引资。组织参加中博会、投洽会等重大经贸活动，深入开展长三角、珠三角、京津冀等先进地区产业招商活动，办好中小企业跨境撮合对接洽谈会。用好第三方招商平台，积极开展中介招商、资本招商、以商招商、产业链招商，再引进一批建链、延链、补链、强链项目。持续扩大对外贸易。积极融入“一带一路”，深度融入“五区”“四路”，加快建设中欧先进制造产业园、优合大罗塘农产品冷链园区，推进德众综合保税区申建工作。谋划举办港澳经贸交流活动，赴欧美举办经贸招商活动，大力培育外贸新增点。

（六）深入推进科技创新，增强高质量发展动力。把创新作为高质量发展的第一动力，壮大创新主体，厚植创新载体，优化创新环境，建设中原科技创新高地。

打造科技创新企业。完善激励企业自主创新政策，强力实施创新龙头企业提升引领、高新技术企业倍增、“科技小巨人”企业培育工程。建立科技企业投资引导基金，持续加强科技贷款工作，引导创新资金资源更多向企业集聚，力争新增高新技术企业20家。深入实施转型先锋行动计划，再引进转化一批中科院科技成果。

建设科技创新平台。深入推进示范区管理体制与人事薪酬制度改革，推动河南理工大学科技园创建国家级大学科技园，加快建设科技总部新城、数字经济产业园等创新载体，推动高新区全国综合评价排名提升。加快十大科技创新平台建设，新增一批工程技术研究中心、企业技术中心、重点实验室、科技孵化器、众创空间等创新平台。深化与国家知识产权局专利审查协作河南中心合作，加快实施专利导航工程，培育一批高价值专利，转化一批优秀专利技术，孵化一批知识产权小微企业，发展一批知识产权密集型产业，带动全市科技创新水平整体提升。

优化科技创新服务。争创国家、省创新型城市和创新型县（市），抓好省“十百千”转型升级创新专项申报实施工作，推动重点产业提质增效。积极参加中国·河南开放创新暨跨国技术转移大会。加快建设科技创新综合服务中心，引进一批国内知名科技服务机构。支持企业、高校、科研院所构建共性技术平台，完善大型科研仪器设备共享机制。

培育科技创新人才。大力实施人才强市战略，完善“引才聚焦”政策体系，加快国际人才城、人才公寓建设，建立分层次人才培育引进机制，突出“高精尖缺”导向，培育引进一批高端科技领军人才（团队）、高素质企业家、高技能人才队伍，努力形成人才综合竞争优势，为高质量发展提供强大智力支撑。

（七）促进区域协调发展，推进城乡高质量建设。坚持把城乡作为整体，科学谋划、统筹建设，全力打造精致城市、品质焦作，着力构建城乡融合、区域协调发展新格局。

加快推进郑焦深度融合。全面落实郑焦融合发展合作协议，启动编制郑焦融合发展全域空间规划，充分释放融合发展的多重效应。打通“大动脉”。围绕建设郑州大都市区门户枢纽城市，加快呼南高铁豫西通道、新焦城际铁路、焦平高速、沿太行高速等项目前期，推进孟州至偃师黄河大桥等项目建设，深入开展詹店黄河大桥、东海大道南延黄河大桥、焦温快速通道南延黄河大桥等项目前期工作，加快打造郑焦综合交通体系。建好“融合区”。加快推进南部产业新城规划建设，编制完成郑焦产业带、沿黄河北岸产业带规划，加快发展高端装备、智能制造、电子信息、生物医药等先进制造业，探索建立郑焦区域协同发展示范园区；有序推进北部生态新城重点开发，积极发展高端会展、文化休闲、健康养生等现代服务业，全力打造郑州大都市区北部现代产业发展新高地。找准“突破点”。启动郑焦融合生态文化旅游专项规划编制，共建郑焦山地生态休闲养生度假游、黄河文化风情游、南水北调生态文化游、中国功夫之旅体验游等精品线路，谋划建立“云台山—嘉应观—陈家沟—少林寺”区域一二三产业融合发展走廊，以文旅融合推动郑焦深度融合发展。同时，深化物流、生态等领域合作，促进人才、科技、教育、医疗、养老等资源共享，推动郑焦深度融合取得新突破，使郑焦融合成为引领焦作崛起的强劲引擎。

全面提升中心城区品质。深入推进“四城联创”，持续完善城市功能，优化城市空间，丰富城市内涵。强力推进十大基础设施项目。一是完成南水北调城区段生态保护带绿化、山体景观等工程，加快“第一楼”和“纪念馆”建设。二是完成李河示范段生态治理工程，实施白马门河、翁涧河、普济河等部分河道生态治理工程。三是完成中原路及绿化带建设，加快推进焦温快速通道（西环路）项目。四是完成东海大道焦辉路至大沙河桥南（除铁路节点外）建设任务。五是推进焦作至荥阳黄河大桥及连接线项目，确保年底前竣工。六是加快南水北调配套水厂建设，苏蔺水厂3月份全面通水，府城水厂7月份建成通水。七是创优太焦铁路建设环境，确保年底前完成线下工程。八是加快大沙河生态治理，完成城区段治理工程，部分区域向市民开放。九是建成新河商务区高压线路归并改造管廊主体，加快新河生态治理和商务中心区建设。十是全面推进棚户区（城中村）改造，确保完成年度任务。扎实开展“四城联创”。大力弘扬社会主义核心价值观，深入开展“思想道德素质、诚信守法、文明风尚”三大提升行动，着力提高市民文明素质，努力打造人民满意的文明城市。大力实施主次干道维修、背街小巷整治等工程，全面整治无主楼院和老旧小区。加快世行贷款绿色交通项目建设，集中供热、燃气普及率分别达到90%、98%。加快推进城市双修项目。完成新华街、花园街特色风貌试点街道建设。提升城市亮化水平，加快完成太极体育中心等夜景工程。加快海绵城市示范区建设，海绵化改造一批中心城区老旧小区，强力推进龙源湖改造、城东体育健身公园等项目。全面提升治理水平。深化城管体制改革，依法梳理城市管理执法权责清单，形成职责清晰、分工明确、权责对等的管理体系，提升城市治理精细化水平。

扎实推进百城建设提质。坚持抓重点、补短板、强弱项，进一步健全完善促进城镇人口集聚的政策措施，推进农业转移人口在城镇落户，推动县域经济集聚化、特色化、差异化发展。围绕全域百城建设提质，写好“四篇文章”、提升“四个品质”、注重“四个统一”、抓好“四项治理”，着力推进城市规划、建设、管理、经营高质量。在全市道路、供水、燃气、污水垃圾处理等领域，加快建设总投资1429亿元的511个提质项目，确保完成投资200亿元。六县（市）建成区公共用水、燃气普及率分别达到90%、92%，生活污水、垃圾处理率分别达到95%、100%，促进城市由外延扩张向内涵提质转变。

大力实施乡村振兴战略。落实乡村振兴战略的总要求，准确把握关键环节，加快实现农业强、农村美、农民富。着力稳定农业生产。全面落实粮食安全行政首长责任制，完成粮食生产功能区和重要农产品生产保护区划定工作，加快建设高标准粮田。积极做好小浪底北岸灌区、西霞院水利枢纽输水及灌区等重大水利工程建设相关工作。调整优化农业结构。合理调整“粮经饲”结构，推进“四优四化”，壮大蒙牛、伊赛、博农等“两牛”龙头企业，培育“育繁推一体化”现代农作物种业集团，推动“四大怀药”规范化种植、规模化经营。强化农村产业支撑。加快绿色食品业转型升级，大力发展乳品、肉制品、方便食品、饮品、怀药和果蔬等产业，增加优质绿色农产品供给。加快建设焦作国家农业科技园区、温县国家现代农业产业园，推动实施中科焦农高效农业示范园、中荷现代设施农业等高效农业项目，促进农村一二三产业融合发展。改善农村人居环境。扎实推进美丽乡村提升工程，深入实施农村人居环境整治三年行动，积极创建“五美庭院”“四美乡村”和“美丽小镇”，重点抓好农村垃圾污水处理、村容村貌提升，实现“全域净、一眼净”。积极推进“四好农村路”建设，完成2865公里年度创建任务。大力开展农村“厕所革命”，完成27万户改厕任务。加强和创新基层政务服务工作，推进政务服务体系向农村延伸。实施乡风文明建设行动，培育文明乡风、良好家风、淳朴民风。

（八）强力保障改善民生，共享高质量发展成果。始终坚持以人民为中心的发展思想，尽力而为、量力而行，完善制度、守住底线，精心做好省市重点民生实事，不断优化公共服务供给。

着力稳定就业创业。把稳就业摆在突出位置，实施失业保险援企稳岗护航、就业援助等系列行动，全力做好高校毕业生、退役军人、城镇就业困难人员、去产能分流职工等重点群体就业工作。持续实施全民技能振兴工程，鼓励支持外出务工人员返乡创业，带动更多农村劳动力就近就地就业。降低创业担保贷款门槛，更好发挥贷款倍增效应。

全面加强社会保障。加快建设全国社会保险标准化先行城市，深入实施全民参保计划，用好助保贷款政策，做好社保基金征缴工作。完善“两定制兜底线”健康扶贫模式，统筹医疗救助与基本医保、大病保险、大病补充保险政策的衔接，提升重特大疾病医疗救助水平。推进残疾人康复教育中心建设，加强残疾人权益保障。提高城乡低保、特困供养、孤儿养育等保障标准，推动农村低保制度和扶贫开发政策有效衔接。

大力发展社会事业。全面提升教育质量，推动城乡义务教育一体化发展，推进学前教育普及普惠、安全优质发展。大力发展职业教育，加快建设“双元”技术技能人才培育改革试点。强力推进市实验幼儿园新区分园、市十二中东部校区、市特教学校学生公寓及康复楼、市技师学院新校区、韩公教育园区等项目规划建设，努力让每个孩子都能享有公平而有质量的教育。推动高等教育内涵发展，支持河南理工大学建设一流学科，提升焦作大学、焦作师专办学水平，加快黄河交通学院新校区项目建设。实施健康素养促进行动，开工市人民医院门急诊大楼及医学研究中心、市中心血站搬迁项目，加快建设市级区域医疗中心。探索太极体育中心市场化管理运营机制，统筹规划建设各类公共服务场馆。完善社会养老服务体系，加快市福利院迁建、城市社区日间照料中心、农村幸福院等项目建设。扎实开展文化惠民活动，提升“百姓文化超市”、农家书屋等文化惠民工程服务水平。建成全省公交优先示范市。加强人民防空建设。实施文物安全基础保障三年行动。实施全民健身计划，大力推进太极拳“六进”，完善城市社区“10分钟体育健身圈”。

推进社会治理创新。认真学习“枫桥经验”，完善提升“334”楼院协商治理模式，深入开展社会矛盾纠纷调处，全面加强基层社会治理。深入开展普法教育，推进法制宣传教育“六进”。健全社会治安防控体系，强力实施“雪亮工程”，高质量推进“一村（格）一警”工作，推动“扫黑除恶”专项斗争向纵深发展，确保社会大局和谐稳定。

同时，积极支持驻焦部队建设，深入开展双拥共建，巩固军政军民团结，全力创建全国双拥模范城。提升退役军人服务管理水平，切实维护退役军人合法权益。加强民族宗教工作基层基础建设，促进民族团结、宗教和睦。支持工会、共青团、妇联等人民团体工作。加强统计调查、外事侨务、地震、对台、气象、档案、史志、援疆等工作。

三、加强政府自身建设

新时代对政府工作赋予了新使命、提出了新要求。全市政府系统要以党的建设高质量推进政府工作高质量、实现发展高质量，坚持以人民为中心，坚持发展第一要务，进一步加强自身建设，努力建设人民满意的服务型政府。

加强政府自身建设，最首要的是政治建设。我们必须始终高举习近平新时代中国特色社会主义思想伟大旗帜，进一步树牢“四个意识”、坚定“四个自信”、做到“两个维护”，坚定不移向党中央看齐，紧跟党中央步伐、紧扣党中央节拍，以政治建设为统领，全面推进政府系统党的建设，始终在政治立场、政治方向、政治原则、政治道路上同以习近平同志为核心的党中央保持高度一致，坚决推动党中央决策、省委部署落地生根、开花结果。要深入推进“两学一做”学习教育常态化制度化，全面贯彻省委“学、严、干”要求，做政治上的明白人，做复兴路上的追梦人。要扎实开展“不忘初心、牢记使命”主题教育，深入推进党风廉政建设，坚决贯彻中央八项规定及实施细则精神，扎实推进以案促改，主动接受纪检监察监督，加强审计和巡视巡察结果运用，强化行政监督和行政执法监督。要深入推进长效制度建设，健全完善公共资金、公共资源、国有资产等监管制度，加快构建不敢腐、不能腐、不想腐的制度体系，打造政府系统良好政治生态。

加强政府自身建设，最根本的是促进发展。发展是硬道理，政府系统必须时刻抓牢发展第一要务。要强化发展意识。各级各部门要深刻认识增长速度换档期、结构优化调整期、发展方式转变期的时代特征，把发展作为应对新情况、解决新问题、迎接新挑战的“金钥匙”，聚精会神搞建设，一心一意谋发展，用发展的思维、改革的办法解决“转型中的困难、前进中的问题、成长中的烦恼”。要把握时代要求。各级各部门要充分认识新时代社会主要矛盾，牢牢把握高质量发展的政治要求和客观目标，全力促进经济结构、产业结构、供给结构、需求结构全面优化调整，坚决不要灰色、黑色GDP，推动我市经济社会持续、健康、高质量发展。要树牢宗旨意识。发展为了人民。政府系统要坚持在发展中主动破解民生难题，提升教育、医疗、住房、社保等保障水平，通过高质量发展满足人民日益增长的美好生活需要，努力实现更高质量、更加平衡、更加充分的发展，让全体人民更广泛更公平共享改革发展成果。

加强政府自身建设，最关键的是勇于担当。习近平总书记强调，有多大担当才能干多大事业。政府工作人员要树立强烈的事业心和使命感，遇到矛盾和问题要敢于负责、勇于担当，切实做到在其位、谋其政、尽其责。要增强担当本领。政府工作人员要提高“八种本领”，防止思想僵化、知识老化、本领弱化，不断增强敢担当、善担当、能担当的素质能力。要积极主动作为。政府系统要弘扬焦裕禄同志的“三股劲”，走出机关、深入基层，在一线推进三大攻坚战、产业转型、项目建设、招商引资、民生实事等中心工作，坚决做到困难面前不推脱、矛盾面前不推诿，敢于担当、敢于较真、敢于碰硬，以钉钉子精神解决实际问题、破解重大难题，有为推动发展。要落实容错机制。严格按照“三个区分开来”，切实把容错纠错机制落到实处，鼓励支持改革创新，宽容探索实践中的失误，激励干部担当作为、干事创业，旗帜鲜明、理直气壮地为依法依规担当的干部撑腰鼓劲。

加强政府自身建设，最重要的是抓好落实。要持续深入开展好“质量、效率、法治、出彩”落实年活动，以“抓铁有痕、踏石留印”的作风，撸起袖子加油干，用实干实现更加精彩。一是进一步提升“质量”。把高质量发展的根本要求贯穿政府工作始终，融入方方面面。要强化质量导向。出台我市高质量考核评价办法，优化考核程序和标准，制订对应工作方案，力争今年全市高质量发展的主要经济指标进入全省“第一方阵”。研究设立高质量发展奖，健全完善评选标准，充分调动各类市场主体提高发展质量效益的积极性。要开展质量提升行动。围绕落实国家和省稳增长、补短板、惠民生等政策措施，制订提升经济运行质量、产业发展质量、投资结构质量、民生保障质量和生态环境质量五大行动计划，逐项开展攻坚，全面提升经济发展总体质量。要做实质量载体。把大抓项目作为推动高质量发展的载体，以推进省市重点项目为抓手，加强跟踪督查，协调解决问题，加快建设进度，确保项目早竣工、早投产、早见效，为经济社会高质量发展提供基础支撑。要确保招商质量。各级各部门主要负责同志要亲自谋划、组织、推动、招引项目，县（市）区政府主要负责同志每月至少安排四分之一时间外出招商。市政府将严格开展项目评审、招商评审，切实增强项目建设和招商引资的实效。要提升督导质量。市政府督查室及相关部门要坚持突出重点、突出成效，对全市中心工作，建立台账、清单管理，全程跟踪、严格考核，确保按期高质量完成。二是进一步提高“效率”。突出问题导向，强化效率意识，打造运转高效、执行力强的效能政府。要提高决策效率。健全完善市政府议事制度，严格遵守重大决策法定程序，推进议事决策的民主化、科学化、规范化、高效化。落实行政首长负责制，各级各部门主要负责同志对职权内的事项，要不回避矛盾、不推诿责任，敢于负责拍板、果断依法决策，坚决杜绝议而不决、决而不行、行而不果。要提高运转效率。各级领导干部要率先垂范、立说立行，当日文、当日结，当日事、当日毕，带头提高效能。加快建立政府系统网上办文办会综合平台，举办公文处理与运转、电子政务运用培训班，推进电子公文传输和无纸化办公，让政府运转更快起来。要提高政事效率。切实克服形式主义、官僚主义新表现，深入开展文山会海、报表材料、督导检查专项清理，确保全市政府系统会议和文件数量减少20%以上，要求基层上报的报表材料、对基层开展的督导检查减少50%以上。要提高服务效率。扎实开展“解难点、疏堵点、除痛点”专项行动，相关职能部门要定期到重点企业开展集中办公，现场听取企业的意见和建议，重点解决项目立项、规划、土地、环评等审批难、审批慢问题；启动全市营商环境评价工作，研究出台营商环境评价体系、评价办法、评价机制，让企业评价政府及部门，促进审批服务效率提升，构建“亲”“清”新型政商关系，打造一流营商环境。三是进一步加强“法治”。坚持依法治国基本方略，把政府工作全面纳入法治轨道。要提高法治能力。严格落实中办、国办《党政主要负责人履行推进法治建设第一责任人职责规定》，各级各部门主要负责同志要认真履行法治建设组织者、推动者和实践者的责任，做尊法学法守法用法的模范。开展政府系统法治专题教育，组织普及法律知识、规范执法行为专项培训，杜绝任性用权。要依法破解难题。加强政府诚信建设，开展“新官不理旧账”大清查活动，对招商引资、企业改制、城市建设等领域遗留问题全面梳理，一事一策、加快解决，合法的践行承诺，不合法的按程序给予明确答复，有争议的积极协商推进，对方已无承担能力的妥善解除协议，依法依规解决好遗留问题。要依法接受监督。自觉接受人大法律监督、工作监督和政协民主监督，聚焦问题整改，加强沟通对接，突出跟踪督导，确保高质量办好人大议案、建议和政协提案。广泛听取民主党派、工商联、无党派人士、人民团体意见，主动接受社会和舆论监督，让权力在阳光下运行。四是进一步追梦“出彩”。全市政府系统要树立强烈的出彩意识，人人“争做出彩焦作人”。各级政府要认真对照确定的目标，深入贯彻各项工作部署，推动经济社会高质量发展，努力在全省争先晋位、更加出彩。政府各部门要跳出焦作看焦作、跳出焦作发展焦作，紧盯本系统先进典型，制订对标方案，紧抓本地突出特色，争取有更多专项在全国全省有位次、出经验。政府全体人员要努力使自己的每件工作都过硬、都出彩，努力用一个个出彩凝成“重彩”，用一件件出彩汇成“精彩”，奋力谱写焦作高质量发展新篇章。

各位代表！思想凝聚力量，奋斗创造未来。让我们以习近平新时代中国特色社会主义思想为指导，更加紧密地团结在以习近平同志为核心的党中央周围，认真贯彻落实省委、省政府和市委的各项部署，以建设全面体现新发展理念示范城市为引领，全力推动经济社会高质量发展，加快建设“四个焦作”，早日跻身全省“第一方阵”，致力打造精致城市、品质焦作，努力在中原更加出彩中出重彩、更精彩，以优异成绩庆祝中华人民共和国成立70周年！
